# Klippel-Trenaunay Syndrome With Atypical Presentation of Small Port-Wine Stain

**DOI:** 10.7759/cureus.28303

**Published:** 2022-08-23

**Authors:** Sama Alazawi, Kevin Wright

**Affiliations:** 1 Dermatology, Naval Medical Center San Diego, San Diego, USA

**Keywords:** capillary venous malformation, pulse dye laser, limb hypertrophy, port-wine stain, klippel trenaunay syndrome

## Abstract

Klippel-Trenaunay Syndrome (KTS) is a rare congenital capillary/venous malformation (CVM) disorder associated with deep soft-tissue swelling. A seven-year-old Caucasian male with a history of Klippel-Trenaunay Syndrome presented to the dermatology clinic for evaluation and treatment of mildly edematous right lower extremity and varicose veins with multiple, small port-wine stains and nodules. Typically, cases of KTS report large port-wine stains, where a multidisciplinary approach is the mainstay for management. However, this case involves a clinical presentation of mild KTS with atypical, *small* port-wine stains on the affected limb.

## Introduction

Klippel-Trenuanay Syndrome (KTS) is characterized by the triad of capillary and venous malformation and limb overgrowth with or without lymphatic malformation [1]. Historically, KTS has had a variety of broad definitions and has been associated with arteriovenous malformations (AVM) known as “Klippel-Trenaunay-Weber” [1]. However, the term “Klippel-Trenaunay-Weber” has evolved into a distinct disorder known as “Parkes Weber Syndrome.” In 95% of patients, KTS exclusively affects the lower extremity, while only 5% are affected in the upper extremity [2]. In rare cases, multiple limbs and body regions like the trunk can be involved. Regardless of whether the upper or lower extremity is affected, KTS usually presents with cutaneous vascular lesions (port-wine stains), varicose veins, soft tissue, and bone hypertrophy, with or without lymphatic anomalies [2]. In most cases, cutaneous vascular lesion (port-wine stain) is discovered at birth, and limb hypertrophy becomes apparent during childhood [2].

## Case presentation

A seven-year-old Caucasian male with a past medical history of KTS presents with his father to the dermatology clinic for evaluation of his right lower limb. The patient’s records revealed that the patient had cutaneous vascular malformation (port-wine stain) on the right knee immediately after birth. Also, an MRI of the patient’s legs revealed venous dilation of the right leg without any involvement of the arteries. At 22 months, the patient’s right leg had significantly increased in size. He was diagnosed with KTS based on the clinical presentation and physical exam of cutaneous vascular malformation with varicose veins and leg hemihypertrophy. Additionally, MRI findings of venous dilation of the right lower extremity supported the diagnosis. The patient also received an MRI of the head/face & neck to evaluate for intracranial involvement. Fortunately, no vascular malformations were found in the head & neck area.

**Figure 1:**
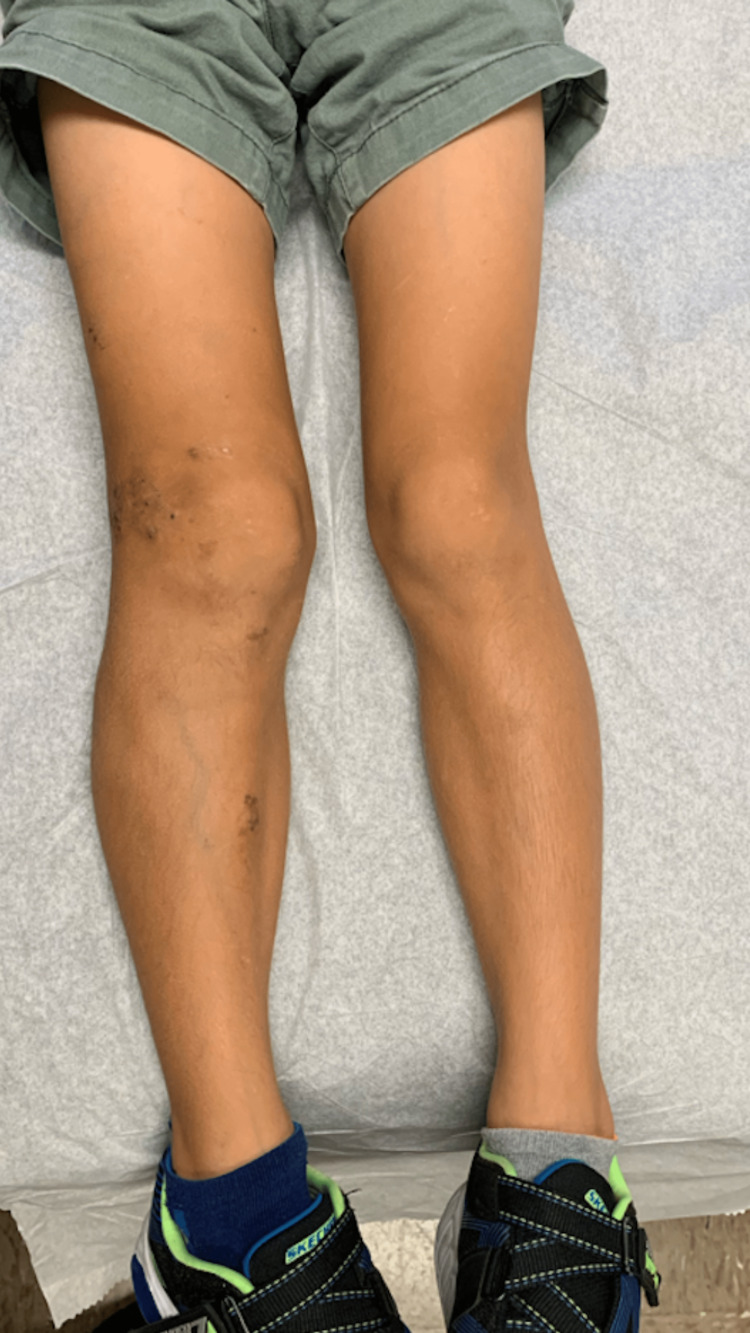
Edematous right leg with multiple scattered violaceous and erythematous plaques

The patient reported tenderness to the right pretibial lesion. To manage his lymphedema, the patient has been using compression stockings. He has a full range of motion of the lower extremities. He has no history of gastrointestinal (GI) bleeding. He was examined by the orthopedics service, who noted the patient to have a mild leg-length discrepancy of about 0.7 cm on X-ray imaging, with increased girth at the right knee. His musculoskeletal exam revealed mild hypertrophy and edema of the right lower extremity (Figure 1), and a normal gait with level hips and shoulder.

The trunk was balanced in sagittal and coronal planes. There was non-painful and full active and passive range of motion of bilateral lower extremities. Based on benign musculoskeletal exam and clinical presentation, orthopedics recommended the patient to continue compression stockings for lymphedema and follow-up in 6 months.

**Figure 2:**
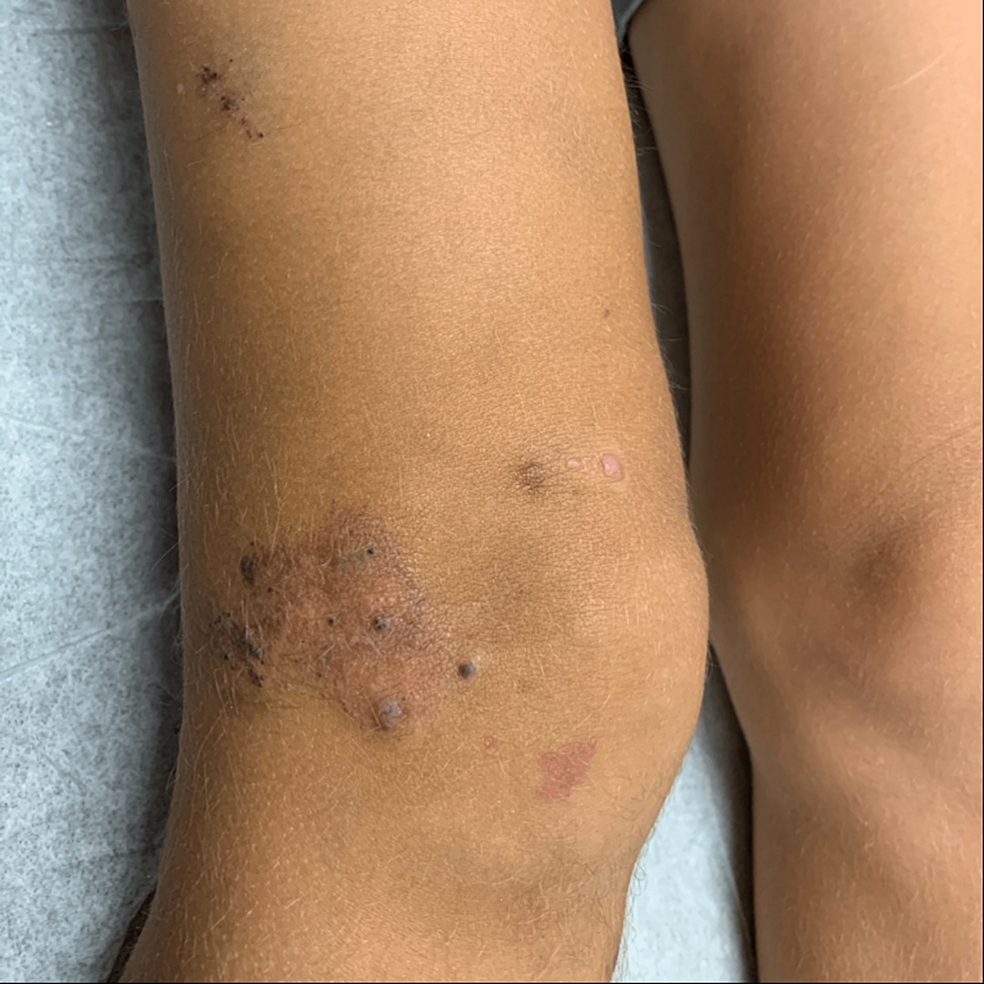
Blue nodules with hemorrhagic crust and surrounding erythematous plaque noted on the lateral right knee consistent with venous malformation and angiokeratomas

**Figure 3:**
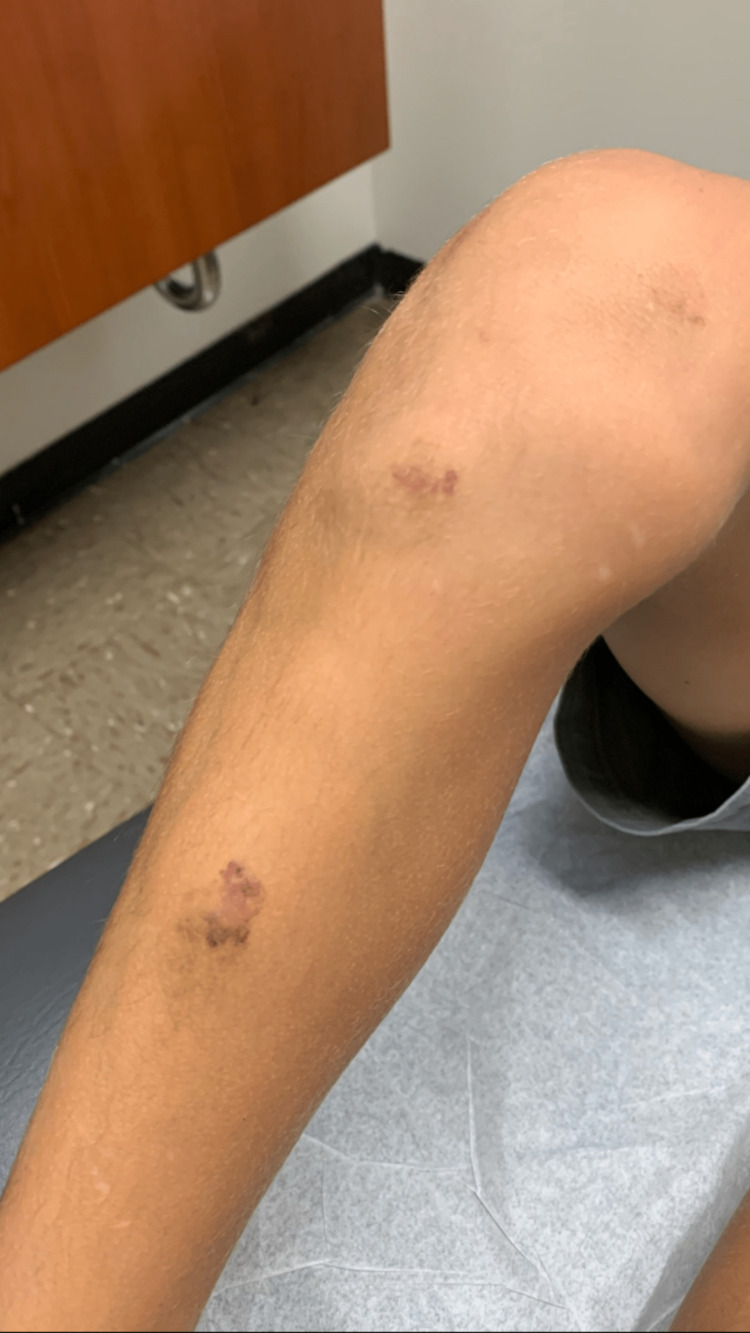
A firm violaceous plaque with tenderness to palpation noted on the right mid-tibial region consistent with phlebolith

**Figure 4:**
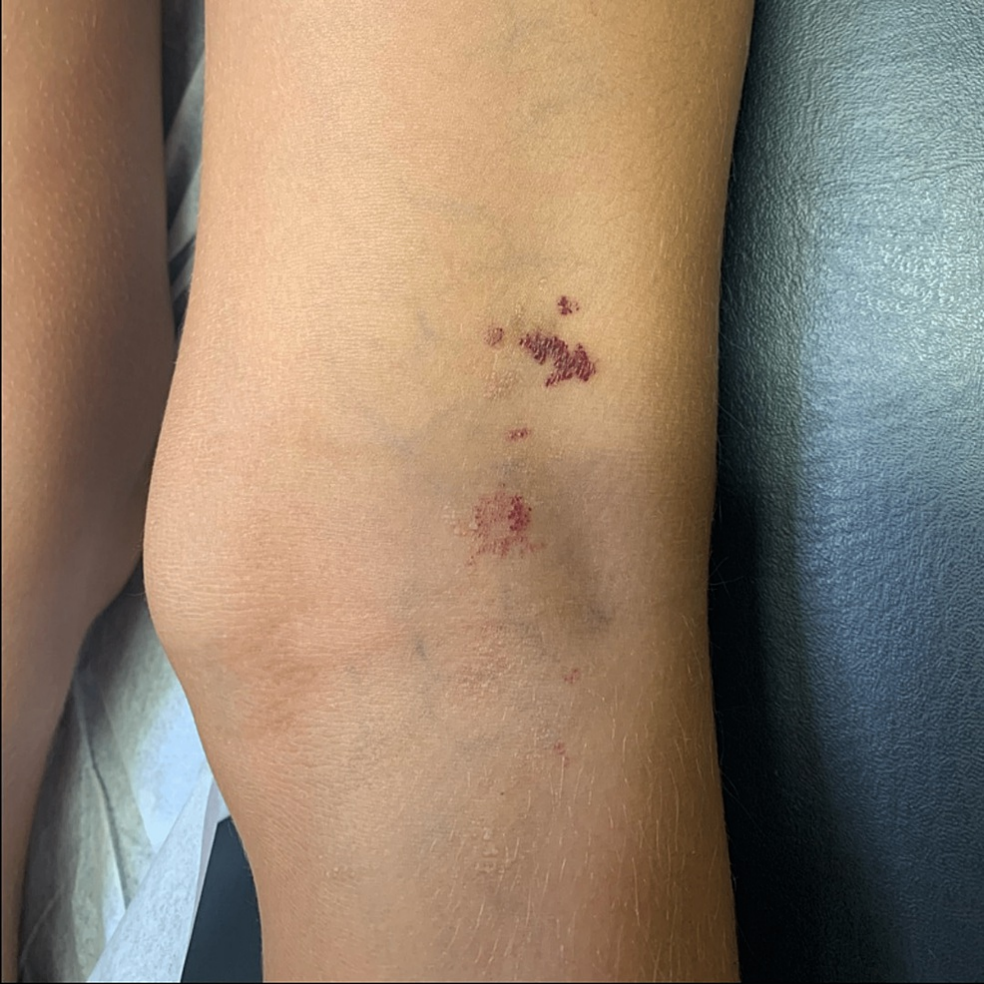
Well demarcated red/violaceous patches with irregular shape noted on the popliteal fossa consistent with geographic port wine stain/ capillary malformation

Skin examination revealed (1) several blue nodules with hemorrhagic crust on the right lower extremity consistent with venous malformation and angiokeratoma (Figure 2); (2) one firm violaceous plaque with tenderness to palpation in the mid-tibial region consistent with phleboliths (Figure 3); and (3) several small (i.e., > 2 cm2) well-demarcated erythematous patches in the right popliteal fossa consistent with capillary malformation (Figure 4). Complete blood count (CBC) and basic metabolic panel (BMP)values were within normal limits. The patient was not taking any oral medications.

Later, the patient received laser treatments at our clinic with Nd (Neodymium):YAG with 1064NM for the angiokeratoma-like lesions and Pulse Dye Laser (PDL) for the port-wine stains. The 1064NM ND YAG was set to 30 ms pulse width, 3 mm spot size, 40/20 DCD, and 130 J/cm2. The PDL was set to 595NM, 6J/cm2, 10MM spot, 0.45 ms pulse width, 2 bars spray. Mild erythema and purpura were noted after the laser therapy session, which usually indicates sufficient laser treatment. The patient tolerated the procedure with mild pain. Retreatment in 4-6 weeks was recommended, but the patient was lost to follow-up. He was also referred to vascular surgery for possible sclerotherapy of phlebolith.

## Discussion

Klippel-Trenaunay Syndrome (KTS) is a rare congenital and sporadic disorder with Capillary Venous Malformations (CVMs) and limb overgrowth. KTS commonly affects unilateral lower extremity. This syndrome has been associated with heterozygote somatic PIK3CA mutation pathway which leads to overexpression of PI3K/mTor pathway, resulting in cell proliferation and angiogenesis [3]. This contributes to tissue and bone overgrowth [3]. Many vascular malformations have clinical overlap with KTS and genomic testing may be needed to make the distinction. KTS is often diagnosed clinically with imaging modalities used to confirm the diagnosis [2]. Ultrasound and MRI of the involved lower extremity are recommended as the initial imaging for suspected KTS [4]. A biopsy is not typically necessary to confirm the diagnosis [4]. This syndrome has an estimated prevalence of two to five per 100,000 people worldwide [5]. KTS appears to be less frequent in individuals of African and Asian descent without gender predilection [3].

Differential diagnoses of other overgrowth syndrome include Parkes Weber Syndrome, which is less likely given the absence of clinical symptoms of AVM such as GI bleeding [1]. There was no evidence of macroglossia or organomegaly on imaging so Beckwith-Wiedemann Syndrome is unlikely [6]. Although Proteus Syndrome can present with capillary, venous, and lymphatic malformations, the patient did not have evidence of scoliosis or pulmonary restrictions [6]. CLOVES (Congenital Lipomatous Overgrowth, Vascular malformations, Epidermal nevi, and Scoliosis/skeletal/spinal anomalies) syndrome usually involves the thorax with lipomatous masses, which are frequently covered with capillary malformations at the cutaneous level, which was lacking in our patient [6]. Additionally, our patient did not have epidermal nevi along the lines of Blaschko, which is usually present in CLOVES syndrome [6]. The absence of macrocephaly excludes PTEN-hamartoma tumor syndrome and megalencephaly-capillary malformation [3].

Venous malformations (VMs) can occur in superficial and deep veins [2]. Superficial venous malformations present with soft-compressible blue masses in soft tissue. Intralesional thrombosis can occur in VMs and can become calcified, leading formation of phlebolith [2]. On the other hand, anomalies of the deep venous system present with leg pain and are often addressed with sclerotherapy by vascular surgery [3]. There is an increased risk of a veno-thromboembolic event (VTE) with frequency ranging from 4%-22% including superficial and deep venous thrombotic events [7]. Pulmonary emboli (PE) have been reported in 4% of KTS patients [7]. Our patient did not have leg pain so we have a low suspicion for VMS involving joints and we also believe that our patient has mainly superficial vein involvement.

Port-wine stains (PWS) occur due to capillary malformation and are usually present as early as birth. Cases of KTS have been reported with large port wine stains and cover a significant surface area of the affected limb [2]. However, this patient only has very small port-wine stains of less than 2 cm. There are two distribution variants of PWS: (1) geographic/continental, which are usually well-demarcated dark red/purple patches; and (2) blotchy/segmental PWS, which are usually ill-defined borders and light red colored patches [8]. This patient has a geographic variant of PWS and is, therefore, more likely associated with lymphatic malformation and tissue overgrowth [8]

For port-wine stains, PDL therapy is the standard of care. However, its efficacy decreases with the presence of deeper nodular lesions. Accordingly, different types of lasers might be warranted to treat superficial venous malformations.

Appropriate parameters for PDL treatment of PWS are as follows: Wavelength of 595nm; Pulse duration 0.45 to 10 msec; Fluence 4.5 to 12 j/cm; Spot size: at least 7mm; and Long pulse 1064nm Nd: YAG is useful for nodular lesions [9].

For the phlebolith and thicker nodules, ND:YAG laser was used. However, this laser usually penetrates 1-3 mm of lesions [10]. Phleboliths are usually thicker than 3 mm and usually do not respond to laser treatment [10]. Thus, he was referred to vascular surgery for possible sclerotherapy, which has been suggested to be a superior treatment modality compared to laser therapy [10]. The overall treatment of phleboliths usually involves a multidisciplinary approach of conservative therapy, laser, sclerotherapy, and surgery [10]. Isolated and asymptomatic phleboliths can be managed with leg elevation, compression stocking, and pain control [10].

Here, our patient did not see improvement from one session of laser therapy, which is expected. Maximum benefits are seen after multiple therapy sessions. Side effects of the PDL therapy include pain, burning, stinging, lack of efficacy, and the potential for blistering, crusting, post-inflammatory hyperpigmentation, hypopigmentation, and scarring [11]. 

KTS is usually treated with a multidisciplinary approach [12]. Physical therapy is recommended for difficulty with mobility and ambulation. Compression stockings and elevation are suggested to reduce blood stagnation and minimize expansion and phlebolith formation [12]. A shoe lift can be used to address mild leg-length discrepancy and surgical intervention may be needed for significant discrepancy [12]. The orthopedic team has determined that our patient does not need a sole insert or surgical intervention at this time for the 0.7 cm limb length discrepancy, but further follow-up is necessary. Typically, if the discrepancy is >1.5 cm, a shoe-lift for the shorter limb can prevent limping and scoliosis [2]. Furthermore, KTS can be managed with vascular interventions, including sclerotherapy [6]. Cutaneous lesions can be treated with laser therapy including a pulse dye laser.

Sirolimus has been suggested in the literature for KTS patients [13]. In one case report of oral sirolimus, which inhibits the downstream motor pathway of PIK3, has yielded a significant improvement of venous lymphatic malformation with control of cutaneous bleeding within 6 months of its use [13]. Thankfully, our patient does not currently have cutaneous bleeding or severe lymphedema, so he does not need this medication at this time. However, Sirolimus may be considered for this patient in the future if his symptoms worsen or he remains refractory to laser therapy. Notably, 27% of sirolimus users experience bone marrow toxicity [14]. Overall, treatment is largely symptomatic and to prevent further complications. For all KTS patients, early referral to dermatology, orthopedics, and vascular surgery for evaluation is imperative to the overall management of KTS symptoms.

## Conclusions

We report a mild case of already rare KTS with atypical presentation of unusually small port-wine stains. The purpose of this report is to improve the early recognition of KTS in the setting of mild clinical presentations. The treatment of KTS is usually conservative and involves a multidisciplinary approach between dermatology, orthopedics, and vascular surgery. For dermatologic care, one session of laser therapy does not usually improve the appearance of port-wine stains. We believe that the maximum benefits of laser therapy are achieved with multiple sessions. Although laser therapy improves the appearance of port-wine stains, multiple sessions of laser therapy are necessary to achieve maximum treatment effect.
